# Phagosome maturation during endosome interaction revealed by partial rhodopsin processing in retinal pigment epithelium

**DOI:** 10.1242/jcs.154757

**Published:** 2014-09-01

**Authors:** Silène T. Wavre-Shapton, Ingrid P. Meschede, Miguel C. Seabra, Clare E. Futter

**Affiliations:** 1 Molecular Medicine Section, National Heart and Lung Institute, Imperial College London, London SW7 2AZ, UK; 2 UCL Institute of Ophthalmology, University College London, London EC1V 9EL, UK; 3 CEDOC, Faculdade de Ciências Médicas, Universidade Nova de Lisboa, 1169-056 Lisbon, Portugal; 4 Instituto Gulbenkian de Ciência, 2780-156 Oeiras, Portugal

**Keywords:** RPE, Phagosome, Rhodopsin

## Abstract

Defects in phagocytosis and degradation of photoreceptor outer segments (POS) by the retinal pigment epithelium (RPE) are associated with aging and retinal disease. The daily burst of rod outer segment (ROS) phagocytosis by the RPE provides a unique opportunity to analyse phagosome processing *in vivo*. In mouse retinae, phagosomes containing stacked rhodopsin-rich discs were identified by immuno-electron microscopy. Early apical phagosomes stained with antibodies against both cytoplasmic and intradiscal domains of rhodopsin. During phagosome maturation, a remarkably synchronised loss of the cytoplasmic epitope coincided with movement to the cell body and preceded phagosome–lysosome fusion and disc degradation. Loss of the intradiscal rhodopsin epitope and disc digestion occurred upon fusion with cathepsin-D-positive lysosomes. The same sequential stages of phagosome maturation were identified in cultured RPE and macrophages challenged with isolated POS. Loss of the cytoplasmic rhodopsin epitope was insensitive to pH but sensitive to protease inhibition and coincided with the interaction of phagosomes with endosomes. Thus, during pre-lysosomal maturation of ROS-containing phagosomes, limited rhodopsin processing occurs upon interaction with endosomes. This potentially provides a sensitive readout of phagosome–endosome interactions that is applicable to multiple phagocytes.

## INTRODUCTION

The retinal pigment epithelium (RPE) is a monolayer of highly polarised cells located between the photoreceptors and the fenestrated endothelium of the choriocapillaris, which forms part of the blood–retina barrier (Burke and Hjelmeland, 2005; Strauss, 2005). The apical surface of the RPE extends long processes that surround the tip of the photoreceptor outer segments (POS). Melanosomes are localised near the apical membrane and in the apical processes (Futter et al., 2004; Gibbs et al., 2004). The RPE performs essential roles for photoreceptor survival, which include absorption of light, protection against photo-oxidation, transport of nutrients, ions and water, secretion of various essential growth factors, and phagocytosis and digestion of shed photoreceptors (Strauss, 2005), the latter being the focus of this paper.

Every day, the distal 10% of the POS are shed as part of a renewal process to maintain their excitability (Young and Bok, 1969; Strauss, 2005). RPE cells phagocytose and digest the POS, and essential substances, such as retinal, are recycled back to the photoreceptors (Thompson and Gal, 2003). The phagocytosis of POS and subsequent phagosome processing by RPE cells potentially provides an opportunity to obtain unique insights into the phagocytic process in a system of great physiological and clinical importance, but also presents considerable experimental challenges. RPE cells are largely postmitotic and so the daily ingestion of POS makes them the most phagocytic cells in the body, each cell digesting in excess of a billion photoreceptor discs in a 70-year human lifespan. This huge phagocytic load eventually takes its toll, and lipofuscin-containing deposits accumulate in all aging RPE and are particularly marked in age-related macular disease (Kennedy et al., 1995). Defects in the engulfment of POS or their subsequent degradation are also associated with inherited retinal degenerative diseases (Gibbs et al., 2003; Gordiyenko et al., 2010; Wavre-Shapton et al., 2013).

Although there are many cultured cell systems used for studies of phagocytosis, the daily synchronised phagocytosis of POS by RPE cells provides a rare opportunity to study phagocytosis in the native environment. The phagocytosed POS have a highly ordered structure of stacked discs that bear high concentrations of visual pigment, allowing changes in morphology and visual pigment processing to be monitored during phagosome maturation. The highly polarised nature of the RPE allows changes in phagosome content to be correlated with changes in phagosome distribution. Limitations of RPE cells in culture have, however, limited progress in understanding phagosome processing in these cells. Most studies have focused on the binding and engulfment of the POS and the machineries involved in these steps. Engulfment of rod outer segments (ROS), which are shed immediately after light onset, requires engagement of αvβ5 integrin and subsequent activation of mer tyrosine kinase (MerTK), annexin A2 and focal adhesion kinase (Feng et al., 2002; Finnemann, 2003; Law et al., 2009; Nandrot et al., 2004). In the Royal College of Surgeons rat, which lacks functional MerTK, ROS fail to be engulfed, leading to rapid retinal degeneration, demonstrating the importance of ROS phagocytosis in protecting the neural retina (Chaitin and Hall, 1983; D'Cruz et al., 2000). Less is known about the subsequent phagosome processing and degradation within the RPE. Phagosomes, once formed, move from the apical to the basal region in order to mature and acquire the capacity to fuse with lysosomes and be degraded (Bosch et al., 1993; Herman and Steinberg, 1982a; Herman and Steinberg, 1982b). A delay in phagosome movement from the apical region to the cell body has been shown in cells that lack myosin VIIa in the shaker-1 mouse model (Gibbs et al., 2003). This is a model for the human disease Usher syndrome 1B, which causes gradual retinal degeneration and hearing and vestibular defects (Petit, 2001). In professional phagocytes, such as macrophages, phagosome maturation is characterised by sequential interactions with the endocytic pathway that involve the acquisition of endocytic markers and are essential for subsequent lysosome fusion (Desjardins et al., 1994; Duclos et al., 2000; Flannagan et al., 2012). Proteomic studies have identified multiple Rab proteins recruited to maturing phagosomes that are likely to regulate interactions not only with different components of the endocytic pathway but also potentially with other intracellular compartments, including the endoplasmic reticulum, Golgi and the autophagic pathway (Gutierrez, 2013). Despite the identification of these panels of Rab proteins that are recruited either to all maturing phagosomes or to specific subtypes of phagosome, the function of the majority of phagosomal Rab proteins, the organellar interactions that they regulate and the functional significance of those interactions remain poorly characterised.

In this study, we follow sequential stages in the processing of phagocytosed ROS by analysing retinal sections from eyes obtained at different times after light onset and by challenging primary RPE cells with isolated POS. We show that the same sequential stages of phagosome maturation occur in primary cultured RPE cells as *in vivo* and that limited proteolysis of rhodopsin occurs before lysosomal delivery and coincides with interaction with the endocytic pathway. Interestingly, we also show here that the same stages of phagosome maturation can be seen in macrophages. Therefore, this powerful technique for identifying sequential stages of phagosome maturation *in vivo* and *in vitro* will impact not only on this clinically relevant ocular system but also on phagosome processing in other cellular systems where *in vivo* analysis is not possible.

## RESULTS

### Loss of the cytoplasmic rhodopsin epitope during phagosome maturation

**Fig. 1.**
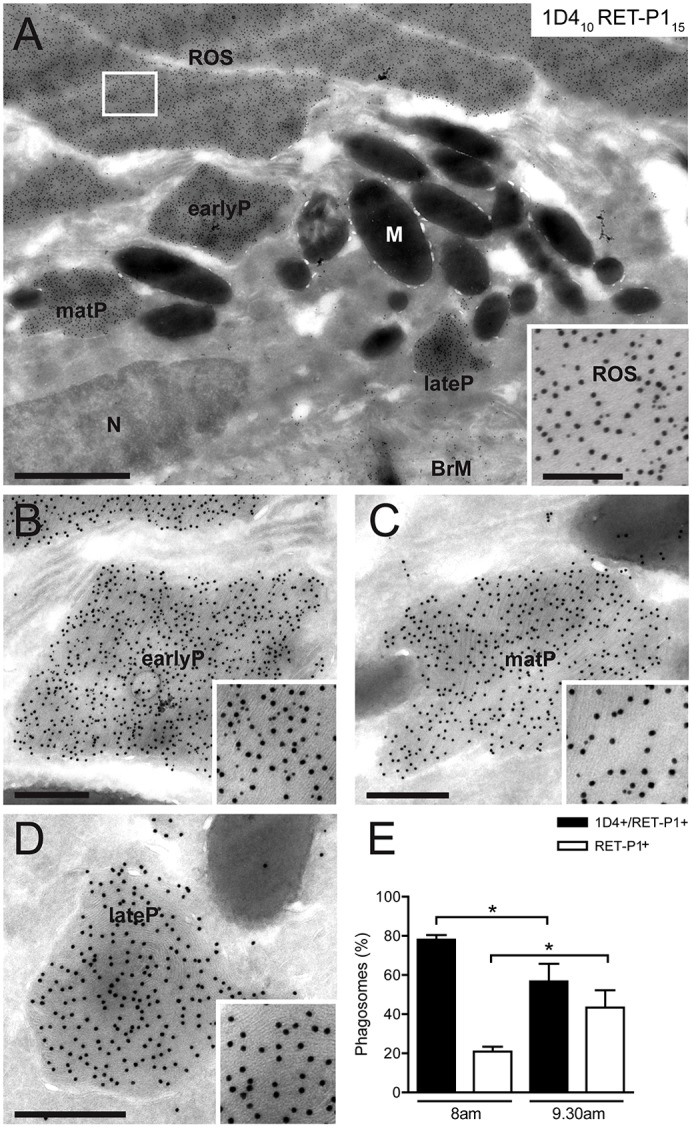
**Loss of the C-terminal cytoplasmic rhodopsin epitope during phagosome maturation.** Immunogold labelling of cryosections of mouse retina collected at 2.5 h after light onset (9.30am). Double labelling of rhodopsin with antibodies against the C-terminal cytoplasmic epitope [1D4; Protein-A–gold (PAG), 10 nm] and N-terminal intradiscal epitope (RET-P1; PAG, 15 nm). (A) Overview of the RPE and POS. The inset shows double labelling in ROS. Higher magnification views of early, maturing and late phagosomes (earlyP, matP or lateP, respectively) in A are shown in B, C and D. N, nucleus; M, melanosomes; BrM, Bruch's membrane. (B) Double-labelled early phagosome. (C) Double-labelled phagosome with low density of 1D4 staining, suggesting that it is a maturing phagosome. (D) Single-labelled phagosome with no 1D4 staining, suggesting it is a mature late phagosome. Scale bars: 1 µm (A), 200 nm (inset in A), 400 nm (B–D). (E) Quantification shows the percentage of total phagosomes at 1 h (8am) or 2.5 h (9.30am) after light onset that are positive for 1D4 and RET-P1 or for RET-P1 only. At each time-point at least 25 phagosomes were analysed in four eyes. Data show the mean±s.e.m.; *P<0.05.

Phagocytosis of shed ROS by the RPE occurs during the first 1–2 h after light onset. We have previously shown 2.5 h after light onset to be a time-point at which both apical and basal phagosomes are present and when phagosomes accumulate if phagosome degradation is impaired (Wavre-Shapton et al., 2013). Thus, at this time, all stages of phagosome processing are likely to be present. It has previously been shown that antibodies against specific rhodopsin epitopes might stain only a subset of ROS-containing phagosomes (Esteve-Rudd et al., 2014; Law et al., 2009). In order to determine whether antibodies against specific rhodopsin epitopes can be used to identify sequential stages of phagosome maturation, we monitored rhodopsin processing by cryo-immuno-electron microscopy using two different rhodopsin antibodies. The RET-P1 antibody binds to the N-terminal intradiscal domain of rhodopsin, whereas 1D4 recognises the C-terminal cytoplasmic domain of the protein. ROS and some phagosomes located in the apical region very close to the ROS (early phagosomes) contained both epitopes (insets, Fig. 1A,B, respectively). Outer segment discs were clearly visible in these 1D4- and RET-P1-positive phagosomes. Interestingly, these specimens also contained phagosomes in the apical region and in the cell body that were strongly positive for RET-P1 staining but contained very little 1D4 cytoplasmic epitope staining (maturing phagosomes) or, more commonly, no 1D4 staining (late phagosomes), as illustrated in Fig. 1C,D, respectively. Despite the scarcity of 1D4 staining, most of these phagosomes contained clearly visible discs. To determine whether phagosomes staining for both rhodopsin epitopes (early and maturing phagosomes) and phagosomes that had lost the cytoplasmic 1D4 epitope (late phagosomes) represented sequential stages in phagosome maturation, the two types of phagosome were quantified at 1 h and 2.5 h after light onset (8am and 9.30am, respectively). As shown in Fig. 1E, at 8am, almost 80% of the phagosomes were double-labelled with RET-P1 and 1D4, compared with 55% at 9.30am. This indicates a progression over time from double-labelled to single-labelled phagosomes during the maturation process.

### Loss of the C-terminal rhodopsin epitope correlates with movement from the apical to the basal region of RPE

**Fig. 2.**
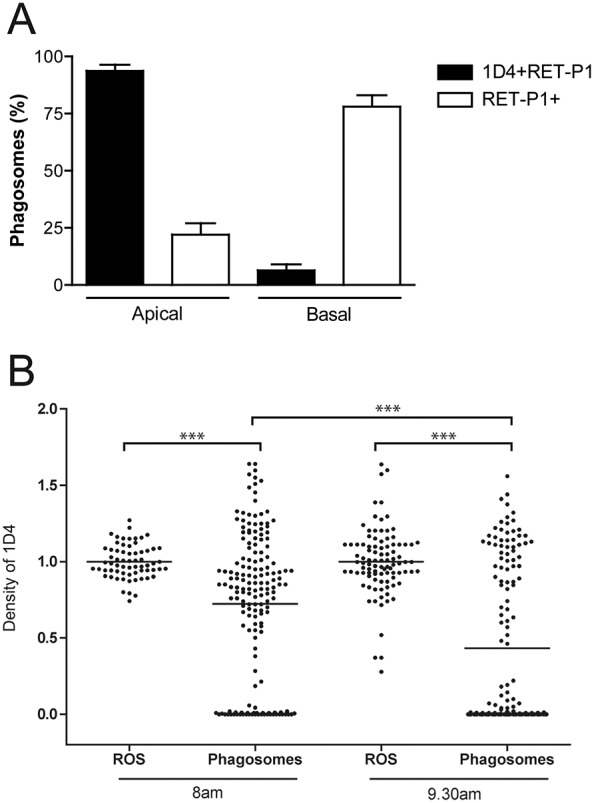
**Synchronised loss of the C-terminal cytoplasmic rhodopsin epitope correlates with phagosome position in the RPE.** (A) The positions of the phagosomes analysed in Fig. 1E were scored as apical or basal depending on whether the phagosome was above or below the tight junctions, respectively. Phagosomes from both time-points were pooled together for this analysis, as their position according to the labelling does not vary with time. Data are expressed as a percentage of total double-labelled phagosomes (black bars) or total single-labelled phagosomes (white bars). A total of 313 phagosomes were analysed in eight eyes. Data show the mean±s.e.m. (B) In the same population of phagosomes as in A, the density of PAG associated with 1D4 was calculated in phagosomes and outer segments. 1D4 density in >60 ROS and >140 phagosomes was calculated in total and normalised to outer segment density in each sample. Four eyes were analysed for each time-point. The thin horizontal black lines show the mean in each sample; ***P<0.0001.

To determine whether the loss of the cytoplasmic rhodopsin epitope correlated with phagosome movement from the apical to the basal region of the cell, the position of phagosomes in the RPE in relation to their labelling for both rhodopsin antibodies was analysed. As shown in Fig. 2A, >90% of early phagosomes (1D4 positive, RET-P1 positive) were apical (at or above the tight junctions), whereas late phagosomes (1D4 negative, RET-P1 positive) could be both apical and basal (below the tight junctions), although most (>75%) were basal. This suggests that the loss of the cytoplasmic epitope of rhodopsin occurs early in the maturation process and might precede movement to the basal region.

### Synchronised loss of the cytoplasmic rhodopsin epitope during phagosome maturation

In order to further analyse the loss of the cytoplasmic epitope of rhodopsin during phagosome maturation, the density of 1D4 staining on phagosomes was determined and normalised to that on the ROS themselves at 1 h (8am) and 2.5 h (9.30am) after light onset. As shown in Fig. 2B, the density of 1D4 staining was very similar on ROS at both time-points; 95% of outer segments had a density between 0.9 and 1.1. By contrast, two main populations of phagosomes were present: the first with a density similar to outer segments, the second with a density of zero. Over time, a shift from the first to the second population was observed, illustrating that these are sequential stages in the maturation process. Very few phagosomes showed an intermediate density (between 0.2 and 0.6), suggesting that the loss of the cytoplasmic epitope of rhodopsin is a very rapid and remarkably synchronised event.

### Loss of the intradiscal rhodopsin epitope occurs on fusion with the lysosome

**Fig. 3.**
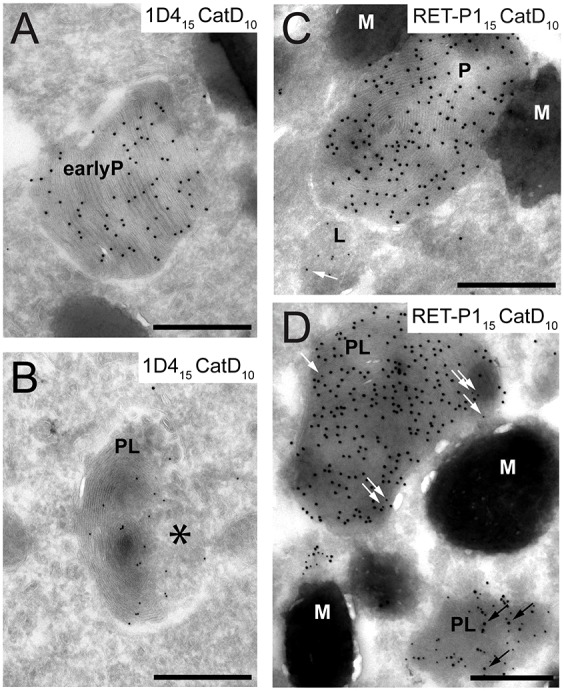
**Loss of the N-terminal intradiscal rhodopsin epitope occurs on fusion with the lysosome.** Immunogold labelling of cryosections of mouse retina collected 2.5 h after light onset (9.30am). (A,B) Double labelling of rhodopsin C-terminal epitope (with 1D4; PAG, 15 nm) and cathepsin D (PAG, 10 nm). (C,D) Double labelling of rhodopsin N-terminal epitope (with RET-P1; PAG, 15 nm) and cathepsin D (PAG, 10 nm). Outer segment discs are visible in all phagosomes and, to some extent, in phagolysosomes. They are less visible in B (asterisk) and they are completely absent from the smaller phagolysosome in D. Scale bars: 500 nm. EarlyP, early phagosome; P, phagosome; M, melanosome; PL, phagolysosome; L, lysosome; black arrows indicate examples of PAG15; white arrows indicate examples of PAG10.

In all phagocytes, phagosomes are eventually degraded upon fusion with lysosomes. In RPE cells, the lysosomal enzyme cathepsin D is very highly expressed (Rakoczy et al., 1999; Yamada et al., 1990; Zhang et al., 2002; Zimmerman et al., 1983), and RPE cells expressing a mutant inactive form of cathepsin D accumulate undigested POS products (Rakoczy et al., 2002), suggesting an important role for this enzyme in POS degradation. We therefore co-stained ultrathin cryosections for cathepsin D and rhodopsin, using 1D4 or RET-P1 (Fig. 3). Interestingly, we found that early phagosomes positive for 1D4 never stained for cathepsin D (Fig. 3A). Cathepsin-D-positive phagosomes or phagolysosomes were negative for 1D4 but could be identified as phagolysosomes (rather than lysosomes) by the presence of ROS discs. However, the discs in phagolysosomes were often morphologically less well defined (Fig. 3B, black asterisk), consistent with some degradation taking place.

Some RET-P1-positive phagosomes were positive for cathepsin D (Fig. 3D); however, the labelling and the morphology of the organelle varied. Phagolysosomes in which outer segment discs were still clearly visible usually had only a few cathepsin D gold particles and were strongly labelled with RET-P1 (PL in upper-left corner in Fig. 3D, white arrows). Phagolysosomes where discs were less visible were more strongly labelled for cathepsin D, whereas RET-P1 staining was weaker (PL in lower-right corner in Fig. 3D, black arrows), indicating a more advanced stage of degradation.

These data suggest that early phagosomes that are positive for 1D4 do not fuse with cathepsin-D-positive lysosomes directly but need some degree of maturation. Loss of the cytoplasmic epitope of rhodopsin is an indicator of this maturation process, which must occur before the phagosome fuses with the lysosomes and full degradation of rhodopsin and outer segment discs can take place.

### Reduced pH does not affect the antigenicity of the cytoplasmic epitope of rhodopsin

**Fig. 4.**
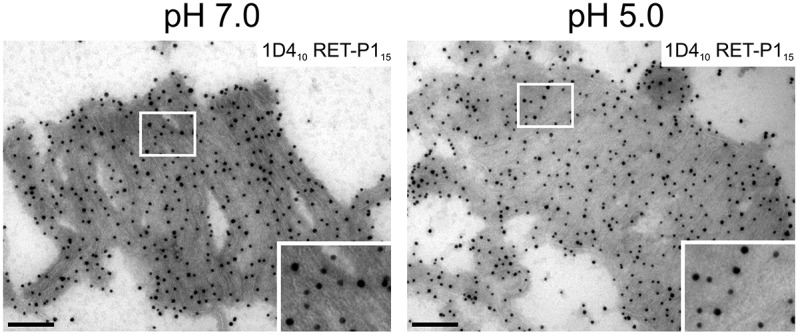
**Reduced pH does not affect binding to the C-terminal cytoplasmic rhodopsin epitope.** POS were incubated in neutral (pH 7.0) or low (pH 5.0) pH solution and were prepared for cryo-immuno-electron microscopy. Ultrathin sections were labelled with 1D4 (PAG, 10 nm) and RET-P1 (PAG, 15 nm). The pH did not affect labelling with the antibody against the cytoplasmic 1D4 epitope. Areas outlined in white are shown at higher magnification in the lower-right corner. Scale bars: 200 nm.

The synchronised loss of the C-terminal rhodopsin epitope raised the possibility that an irreversible conformational change or post-translational modification of rhodopsin in the maturing phagosome might lead to loss of this epitope. Alternatively, a protease present on the ROS themselves might become activated upon phagosome maturation. The most likely activator of any of these changes would be the progressive lowering of the luminal pH of the phagosome that has been shown to occur in other systems (Deguchi et al., 1994; Horwitz and Maxfield, 1984; Kielian and Cohn, 1980). We therefore isolated porcine POS, incubated them in low pH solution (pH 5.0) and determined the effects on C-terminal rhodopsin epitope antibody binding by cryo-immuno-electron microscopy. As shown in Fig. 4, the binding of C-terminal 1D4 and N-terminal RET-P1 antibodies was unaffected by incubation in low pH solution, suggesting that the loss of cytoplasmic 1D4 epitope is not caused by a lowering of pH within the phagosome.

### Loss of the C-terminal cytoplasmic rhodopsin epitope also occurs in cultured RPE cells challenged with isolated POS

**Fig. 5.**
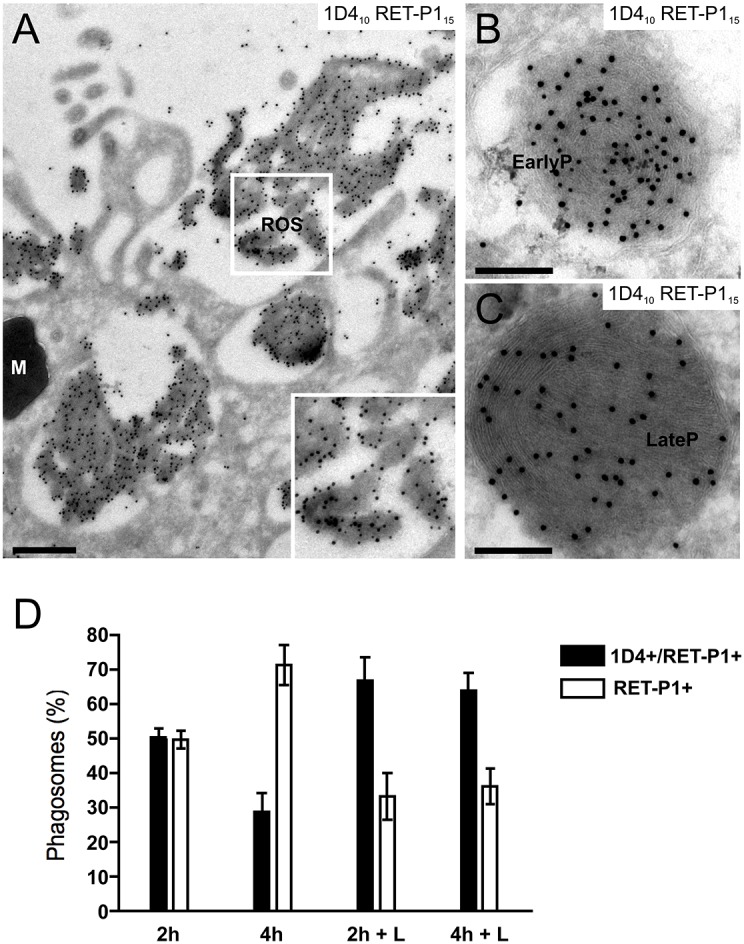
**Partial proteolytic processing of the C-terminal rhodopsin epitope during phagosome maturation in porcine RPE cells.** Monolayers of primary porcine RPE cells on Transwell® membrane inserts were challenged with POS apically for 1 h, washed to remove unbound POS, chased for 2 h or 4 h at 37°C and processed for cryo-immuno-electron microscopy. Ultrathin sections were double labelled for rhodopsin with antibodies against the C-terminal epitope (1D4; PAG, 10 nm) and N-terminal epitope (RET-P1; PAG, 15 nm). (A) An overview of RPE cells and ROS. The inset shows double labelling in ROS. (B) A double-labelled early phagosome with same density of both gold particles as seen on ROS. (C) A single-labelled late phagosome lacking the cytoplasmic 1D4 epitope. Scale bars: 500 nm (A), 200 nm (B,C). M, melanosome; EarlyP, early phagosome; LateP, late phagosome. (D) Quantification shows the percentage of total phagosomes that are positive for both 1D4 and RET-P1 or for RET-P1 only after 2 h and 4 h of chase, in the presence or absence of the protease inhibitor leupeptin (L). Data show the mean±s.e.m. (three independent experiments).

To further investigate the mechanisms underlying the loss of the cytoplasmic rhodopsin epitope, it was necessary to develop a system of cultured RPE cells. As insufficient numbers of cultured mouse RPE cells could be obtained for cryo-immuno-electron microscopy, we developed an *in vitro* assay using primary porcine RPE cells challenged with porcine isolated POS. In order to mimic the *in vivo* situation as closely as possible, primary cells were used only after a single passage and were cultured on Transwell® membrane inserts for 5–10 days in the presence of low serum. Under these conditions, the cells developed a transepithelial resistance of 160–300 Ωcm^2^, and conventional electron microscopy showed that they had the characteristics of polarised RPE cells, with apical processes, melanosomes localised close to the apical membrane and mitochondria around the basolateral border (supplementary material Fig. S1A). To mimic the *in vivo* phagocytosis process as closely as possible, purified POS (supplementary material Fig. S1C) were sonicated for 10 min before addition to the cells. After sonication, the size of the isolated POS (supplementary material Fig. S1D) resembled that of ROS engulfed by RPE *in vivo* (supplementary material Fig. S1B, asterisk). Furthermore, >90% of the isolated porcine POS stained strongly with both 1D4 and RET-P1 antibodies (data not shown), indicating that the majority of this preparation is composed of ROS. To determine whether the same stages of phagosome maturation identified *in vivo* could be identified using our *in vitro* system, cultured porcine RPE cells were challenged with porcine POS for 1 h and chased for 2 h or 4 h (Fig. 5A–C). After 2 h of chase, ∼50% of RET-P1-positive phagosomes were also positive for 1D4 (Fig. 5B,D). After 4 h of chase, most (>70%) of the RET-P1-positive phagosomes were negative for 1D4 (Fig. 5C,D). Thus, there was a progression over time from double-labelled to single-labelled phagosomes that occurred over a similar timescale to that observed on retinal sections, suggesting that phagosome maturation in cultured cells proceeds through similar stages to that occurring *in vivo*.

### Loss of the C-terminal cytoplasmic epitope of rhodopsin is sensitive to leupeptin

To determine whether loss of the cytoplasmic 1D4 epitope occurs through proteolysis of rhodopsin, the phagocytosis assay was also performed on cells that were pre-incubated with leupeptin, a cysteine, serine and threonine protease inhibitor. This inhibitor was present throughout the whole course of the assay. As shown in Fig. 5D, there was an increased percentage of double-positive phagosomes (early phagosomes) in the presence of leupeptin, and a twofold reduction in the percentage of single-positive phagosomes (late phagosomes) after 4 h of chase. This demonstrates that loss of the cytoplasmic 1D4 epitope during phagosome maturation occurs through proteolytic cleavage of a C-terminal portion of the molecule. Leupeptin only partially inhibited the loss of 1D4, probably because this membrane-impermeable inhibitor relies on endocytosis to enter the endocytic pathway and an insufficient concentration might have reached the appropriate endocytic compartment.

### Loss of 1D4 staining occurs upon interaction of phagosomes with a pre-lysosomal compartment of the endocytic pathway

**Fig. 6.**
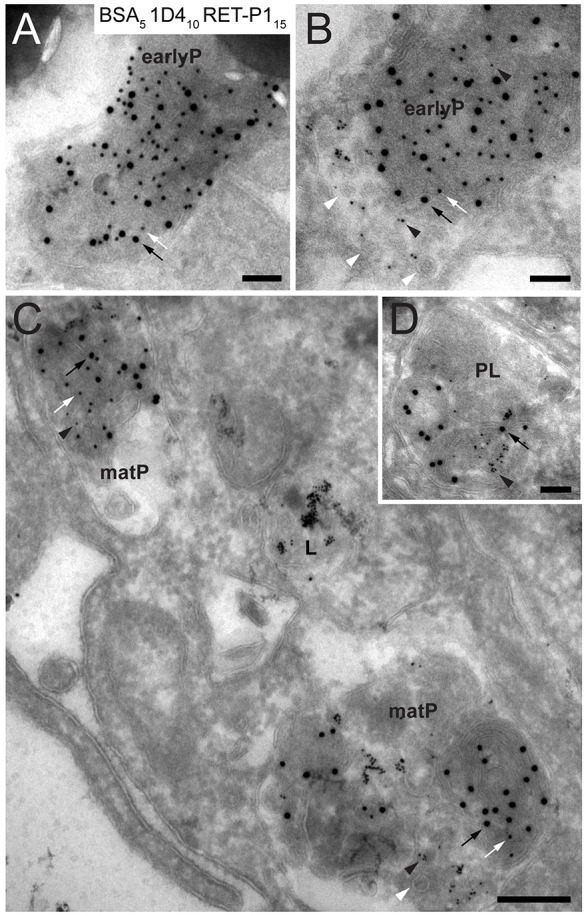
**Early phagosomes interact with a pre-lysosomal compartment of the endoytic pathway in primary porcine RPE cells.** Monolayers of primary porcine RPE cells on Transwell® membrane inserts were incubated in the lower chamber with 5-nm BSA–gold for 3 h prior to the addition of POS, and BSA–gold was maintained throughout the duration of the experiment. Cells were then challenged with POS from the apical chamber for 1 h, washed to remove unbound POS, chased for 2 h at 37°C and processed for cryo-immuno-electron microscopy. Ultrathin sections were double labelled for rhodopsin with antibodies against the C-terminal epitope (1D4; PAG, 10 nm) and N-terminal epitope (RET-P1; PAG, 15 nm). (A) A double-labelled early phagosome (earlyP) that contains no 5-nm gold particles. (B) A double-labelled early phagosome containing monodisperse BSA–gold (black arrowheads) and ILVs (white arrowheads) at one pole, suggesting recent fusion of the early phagosome with a multivesicular endocytic compartment. (C) Maturing phagosomes (matP) containing 5-nm BSA–gold. Note that the density of PAG 10 nm is greatly reduced compared with that of the early phagosome shown in A and B. A lysosome (L) containing aggregated 5-nm gold particles can be readily identified. (D) Phagolysosome (PL) containing PAG 15 nm only and aggregated 5-nm gold particles. Scale bars: 100 nm (A,B,D), 200 nm (C). Black and white arrows indicate rhodopsin labelling with RET-P1 and 1D4, respectively.

As loss of the cytoplasmic rhodopsin epitope occurred before lysosomal fusion, the endosome was a likely source of the protease that cleaves the cytoplasmic rhodopsin epitope during phagosome maturation. Although phagosome–lysosome interactions are relatively easy to measure through the acquisition of lysosomal enzymes like cathepsin D, most studies of interaction with the endocytic pathway have relied on measuring the acquisition of endosomal markers or the acquisition of overexpressed Rab proteins. We sought a more direct measure of phagosome–endosome interactions, and took advantage of the polarised nature of RPE cells cultured on Transwell® membrane inserts that allow the phagocytic and endocytic pathways to be loaded from opposite sides of the monolayer. The presence of particles endocytosed from the basal surface in phagosomes containing ROS taken up from the apical surface would indicate phagosome–endosome interaction. This approach relies on probes endocytosed from the basal surface having access to those parts of the endocytic pathway that can potentially interact with phagosomes. In polarised MDCK cells, probes endocytosed from basal and apical surfaces have been shown to meet in a common apical recycling endosome (Apodaca et al., 1994; Futter et al., 1998). To determine whether this would also be the case in the RPE, cells were incubated with 10-nm bovine serum albumin (BSA)–gold and fluid-phase horseradish peroxidase (HRP) for 2 h from opposite chambers. The HRP reaction product and BSA–gold particles colocalised in vacuoles with the morphological characteristics of endosomes – these vacuoles were electron luscent and contained discrete intraluminal vesicles (ILVs) (supplementary material Fig. S2A,B insets). These pre-lysosomal compartments could be clearly distinguished from lysosomes where the two probes also colocalised because the lysosomes were electron dense, had multiple membranous content and the gold particles within the lumen were aggregated, indicating that they were contained within a degradative compartment (supplementary material Fig. S2A, inset). Having established that RPE cells, in common with MDCK cells, have a common apical recycling endosome that can be accessed from the basal surface of polarized RPE, cells cultured on Transwell® membrane inserts were incubated with 5-nm BSA–gold in the basal chamber for 3 h prior to challenging the cells with POS in the apical chamber, as described above. Cells were then processed for cryo-immuno-electron microscopy and subsequently labelled for rhodopsin with antibodies against both C-terminal cytoplasmic (1D4) and N-terminal intradiscal (RET-P1) epitopes. Gold-loaded elements of the endocytic pathway that did not have phagocytic content could be readily identified. Monodisperse BSA–gold particles could be found in endocytic vacuoles, most of which were multivesicular endosomes/bodies (MVBs), whereas aggregated BSA–gold could be found in electron dense lysosomes (supplementary material Fig. S3A). POS added to the upper chamber contained no gold particles (supplementary material Fig. S3D,E), confirming that the monolayer remained intact during the course of the experiment. Within the cells, the maturing phagosomes could be divided into roughly four categories: (i) double-positive early phagosomes that contained no BSA–gold, indicating that they had not yet fused with the endocytic pathway (Fig. 6A); (ii) BSA–gold-containing double-positive phagosomes, where, in some cases (e.g. Fig. 6B), one pole of the phagosome contained BSA–gold and ILVs largely separate from the double-positive ROS discs, suggesting recent fusion between the phagosome and MVB; (iii) phagosomes that had lost most of the C-terminal 1D4 epitope where the endocytosed gold particles and phagocytosed ROS are mixed (maturing phagosomes in Fig. 6C) – importantly, in this category, the BSA–gold particles remained monodisperse, confirming that they were derived from a pre-lyosomal endocytic compartment; and (iv) phagolysosomes containing only the N-terminal (RET-P1) epitope with aggregated 5-nm gold particles, indicating that the phagosome had fused with the lysosome (Fig. 6D). These data show that loss of the 1D4 epitope only occurs after interaction of the phagosome with the endocytic pathway.

### Phagosome–endosome–melanosome interactions

Examination of retinal sections reveals frequent profiles suggesting a close association of phagosomes with melanosomes. Although apical melanosomes were observed in the vicinity of early apical phagosomes, melanosome–phagosome interactions were mainly in the cell body with mature phagosomes that had lost the 1D4 epitope (see examples in Figs 1 and 3). We found that, in cultured porcine RPE where the endocytic pathway was loaded with BSA–gold from the basal surface, vacuoles could be observed containing melanin particles, monodisperse BSA–gold and ILVs, suggesting MVB–melanosome fusion (supplementary material Fig. S3B). Furthermore rhodopsin-containing phagosomes could also be observed associated with melanosomes, but only if they contained endocytosed BSA–gold, indicating that they had already fused with endosomes and had begun to lose 1D4 staining (supplementary material Fig. S3C).

### Sequential stages of phagosome maturation can also be identified in macrophages

**Fig. 7.**
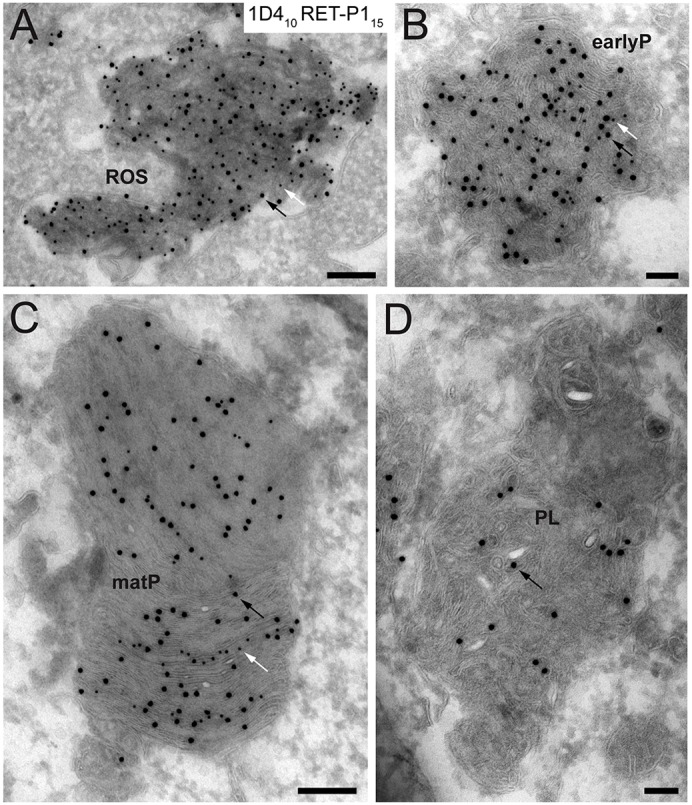
**Sequential stages of phagosome maturation can also be identified by monitoring rhodopsin in macrophages challenged with POS.** J774A.1 macrophages seeded onto Transwell® membrane inserts were challenged with POS for 1 h, washed to remove unbound POS, chased for 2 h at 37°C and processed for cryo-immuno-electron microscopy. Ultrathin sections were double labelled for rhodopsin with antibodies against the C-terminal epitope (1D4; PAG, 10 nm) and N-terminal epitope (RET-P1; PAG, 15 nm). (A) Double-labelled ROS at the plasma membrane. (B) A double-labelled early phagosome (earlyP). (C) A maturing phagosome (matP) containing only very little 1D4 on some discs. (D) A phagolysosome (PL) containing disorganised discs where RET-P1 has started to be degraded. Scale bars: 200 nm (A,C), 100 nm (B,D). Black and white arrows indicate rhodopsin labelling with RET-P1 and 1D4, respectively.

A range of different surface molecules are used by phagocytes to recognise and engulf particles, although there are also some common components of the core phagocytic machinery. Less is known about the molecular regulation of phagosome maturation, but proteomic studies of maturing phagosomes suggest that a combination of core and cell-type-specific and particle-type-specific components are likely to be used. Our finding that proteolytic processing of rhodopsin occurs following fusion with endosomes suggests that rhodopsin processing might prove to be a very sensitive readout of a phagosome–endosome interaction that is central to phagosome maturation in multiple phagocytes. Therefore, we investigated whether the C-terminal rhodopsin processing also occurred during phagosome maturation in the macrophage cell line J774.1, when challenged with POS. After a 1-h pulse and a 2-h chase, cryo-immuno-electron microscopy using both rhodopsin antibodies, 1D4 and RET-P1, showed that the cytoplasmic 1D4 epitope was lost before the intradiscal RET-P1 epitope (Fig. 7), as observed in RPE cells. Loss of the cytoplasmic epitope was rapid in the macrophage cell line, such that most phagosomes had a lower density of cytoplasmic 1D4 staining than the ROS before engulfment (compare Fig. 7A with Fig. 7B). Even in phagosomes that had largely lost the 1D4 epitope, individual discs could sometimes be observed that retained 1D4 staining (Fig. 7C). As with RPE cells, loss of the intradiscal RET-P1 epitope was only observed in phagosomes that appeared to have fused with electron-dense lysosomes and had lost morphologically identifiable discs (Fig. 7D).

## DISCUSSION

Phagosome maturation has been analysed in numerous model systems and is characterised by the sequential acquisition of endocytic markers, including multiple Rab proteins and Rab effectors (eg. EEA1 and Vps34) (Desjardins et al., 1994; Fratti et al., 2001; Gutierrez, 2013; Vieira et al., 2003). This ordered series of events can be circumvented by certain microorganisms and parasites to allow escape from degradation (reviewed in Vergne et al., 2004), and the speed and regulation of phagosome maturation varies amongst different phagocytes and different phagocytic cargoes. Comparatively little is known about the sequence of events leading to phagosome maturation in the RPE, despite the enormous phagocytic burden of these cells and the accumulation of the products of incomplete phagosome degradation that occurs with age and in some retinal degenerative diseases. The relatively synchronised burst of ROS phagocytosis that occurs after light onset allows phagosome maturation to be analysed in RPE cells in their native environment, using mouse retinal sections.

**Fig. 8.**
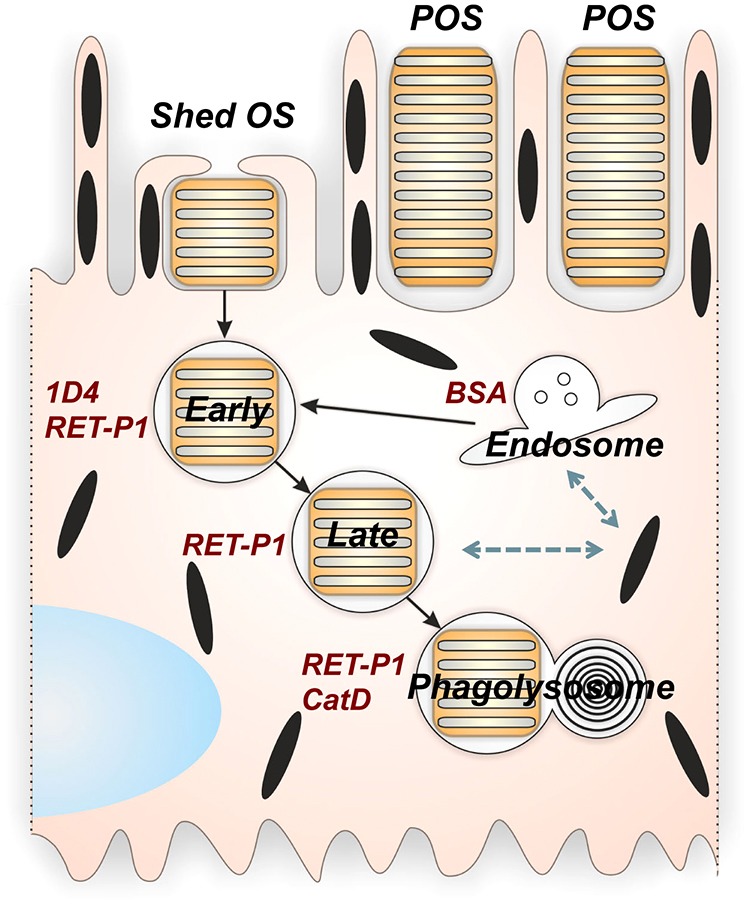
**Sequential stages of phagosome maturation in the RPE.** Early phagosomes are positive for the C-terminal cytoplasmic 1D4 and N-terminal intradiscal RET-P1 epitopes. As the phagosomes mature, 1D4 staining is lost and mature phagosomes are only positive for RET-P1. Maturing phagosomes also show little staining for the lysosomal protein cathepsin D. After fusion of the mature phagosome with the cathepsin D (CatD)-positive lysosome (phagolysosome), RET-P1 staining is lost. Loss of the C-terminal 1D4 epitope occurs only after interaction with endosomes. Profiles indicative of an interaction of melanosomes with both maturing phagosomes and multivesicular endosomes have also been observed.

In this study, we use an approach that does not rely on the acquisition of endocytic markers by the maturing phagosome. Instead, we focused on the phagosome content and used cryo-immuno-electron microscopy to simultaneously analyse rhodopsin processing and phagosome content. Immuno-electron microscopy is unaffected by melanin quenching, which limits immunofluorescent analysis of the RPE, and the resolution of immune-electron microscopy allows the ready distinction between surface and internalised ROS and between apical and basal phagosomes. Using this approach, we have shown that sequential stages in phagosome maturation can be identified (Fig. 8). Early phagosomes stain for both intradiscal and cytoplasmically exposed rhodopsin epitopes. During phagosome maturation in RPE cells *in situ*, a cytoplasmically exposed C-terminal rhodopsin epitope is lost before fusion with the lysosome, allowing the identification of a second ‘mature’ stage in phagosome maturation that retains the intradiscal N-terminal rhodopsin epitope. Loss of the intradiscal epitope occurs only upon fusion with the lysosome and subsequent degradation of the ROS discs, allowing mature phagosomes to be distinguished from phagolysosomes.

To analyse the mechanism of pre-lysosomal rhodopsin processing it was necessary to analyse phagosome maturation in cultured RPE. Although cultured RPE cells isolated from mouse eyes have been used for phagocytosis studies (Gibbs et al., 2010; Gibbs et al., 2003; Nandrot et al., 2007), insufficient cell numbers could be isolated for immuno-electron microscopy experiments. We therefore developed a system using RPE cells isolated from porcine eyes from which ROS could also be isolated. Our system not only generates monolayers of polarised differentiated RPE cells but, most importantly, cells were able to reproduce accurately the *in vivo* process of phagocytosis in terms of phagosome maturation, i.e. the sequential loss of the cytoplasmic rhodopsin epitope before fusion with lysosomes. In addition, the timing of phagosome maturation was very similar to the process *in vivo* (2–4 h), in contrast to a previous study that reported a slower phagosome degradation (12–24 h) in cultured RPE cells (Gordiyenko et al., 2010).

How is the cytoplasmic C-terminal rhodopsin epitope lost? Most phagosomes have either the same density of 1D4 as the ROS themselves or have lost the epitope completely, suggesting a remarkably synchronised process. In other cell types (Deguchi et al., 1994), and in RPE cells (Gordiyenko et al., 2010), there is a progressive lowering of luminal pH as the phagosome matures. This could induce a conformational change in the rhodopsin C-terminus or could even activate a proteolytic enzyme within the ROS themselves, leading to a rapid and synchronised loss of the cytoplasmic epitope. However, we have shown that 1D4 binding to the cytoplasmic epitope of rhodopsin in isolated ROS is unaffected by reduced pH. Alternatively, loss of the cytoplasmic rhodopsin epitope could occur through post-translational modification, such as phosphorylation. Although a partial reduction in the strength of 1D4 binding occurs upon phosphorylation of the C-terminus of rhodopsin (Molday and MacKenzie, 1985), this is unlikely to explain the complete loss of 1D4 binding in the maturing phagosome. Treatment of RPE cells with the protease inhibitor leupeptin substantially reduced the extent of the loss of the cytoplasmic epitope, indicating partial proteolysis of rhodopsin.

Sequential fusion of phagosomes with two different populations of cathepsin-D-positive lysosomes has previously been indicated by Bosch et al. (Bosch et al., 1993). Loss of the cytoplasmic rhodopsin epitope occurs before fusion with cathepsin-D-positive lysosomes and so is not a result of sequential fusion with multiple lysosomal populations. This implicates the endocytic pathway as the source of the protease that cleaves the rhodopsin C-terminus. Interactions between maturing phagosomes and endosomes have not previously been demonstrated in RPE cells. In this study, we have directly measured phagosome–endosome interaction by demonstrating the mixing of phagosome and endosome content, and we have shown that endocytosed gold particles appear in early phagosomes before loss of the cytoplasmic rhodopsin epitope in cultured RPE cells. This strongly suggests that a protease that cleaves rhodopsin is delivered from the endocytic pathway to the maturing phagosome and, thus, cleavage of the C-terminus of rhodopsin is a reporter of phagosome–endosome interaction. The identity of this protease is not yet known, but newly synthesised lysosomal enzymes are delivered to early endosomes through the mannose 6-phosphate receptor (Ghosh et al., 2003; Hirst et al., 1998), although some require lysosomal processing to become active. However, pre-lysosomal proteolytic processing of endocytosed molecules has been reported (Renfrew and Hubbard, 1991), suggesting that some proteases within endosomes are active (Blum et al., 2013).

Our demonstration that a similar processing of rhodopsin occurs in ROS-containing phagosomes in macrophages suggests that loss of the C-terminus of rhodopsin could be a simple, direct and highly sensitive readout of phagosome–endosome interactions applicable to multiple phagocytes. Much of our current information about phagosome–endosome interactions is derived from the acquisition by the phagosome of proteins known to predominantly associate with the endocytic pathway, coupled with knowledge of the function of those endocytic proteins in transport within the endocytic pathway. Cytoplasmic rhodopsin processing will provide a means to directly test the function of these components of the endocytic transport machinery in phagosome–endosome interaction and help to elucidate the nature of phagosome–endosome interactions. It is currently not clear whether these interactions occur via a ‘kiss and run’ type mechanism, as has been described for interactions between endosomes and lysosomes (Bright et al., 2005) or through tubular connections, as have been observed in macrophages between phagosomes and late endosomes and lysosomes (Harrison et al., 2003). In cultured RPE cells, we observed profiles suggestive of full fusion between MVBs and phagosomes. We also observed profiles indicative of fusion between MVBs and melanosomes, an interaction that, to our knowledge, has not previously been described. MVBs form part of the melanosome biogenesis pathway (Raposo et al., 2001), but melanosome biogenesis in mammalian RPE cells is primarily confined to a short window in embryonic life (Lopes et al., 2007) and so, under the conditions of culture used in this study, melanosome biogenesis does not occur. Thus, the profiles that we observed containing endocytosed probes, ILVs and melanin presumably do result from interaction between MVBs and mature melanosomes. Melanosomes are lysosome-related organelles and, in RPE cells, and to some extent in melanocytes, share constituents with lysosomes (Lopes et al., 2007; Raposo and Marks, 2002). It is possible therefore that the molecular machinery that tethers MVBs to lysosomes and promotes their fusion could operate to promote MVB–melanosome fusion in RPE cells, although the function of this fusion is unclear. The function of interaction between melanosomes and phagosomes is also not clear, although evidence of a link between the phagocytic pathway and melanosomes has previously been obtained *in vivo* (Thumann et al., 1999). The presence of lysosomal enzymes in RPE melanosomes raises the possibility that melanosomes might contribute to the degradation of phagosome content, although phagosome–melanosome interactions might also provide protection against oxidative stress induced by phagocytosis and/or against the gradual accumulation of harmful products of phagocytosis, such as lipofuscin.

Our demonstration of the synchronised partial proteolytic processing of rhodopsin provides an exquisitely sensitive readout of phagosome maturation both *in situ* and in cultured cells. It will allow further characterisation of the molecular machinery regulating phagosome processing. Furthermore, it will aid in the identification of defects in phagosome maturation and degradation in aging (Katz and Robison, 1984) and in mouse models of retinal diseases such as choroideremia and Usher's syndrome 1B, which have been shown to involve phagosome degradation defects (Gibbs et al., 2003; Gordiyenko et al., 2010; Wavre-Shapton et al., 2013).

## MATERIALS AND METHODS

### Antibodies and staining reagents

For rhodopsin labelling, RET-P1 and 1D4 antibodies were purchased from Abcam (Cambridge, UK). Antibody against cathepsin D was purchased from Millipore (Billerica, MA). For immune-electron microscopy studies, a rabbit anti-mouse-IgG bridging antibody and Protein-A–gold (PAG) were purchased from Dako (Glostrup, Denmark) and CMC, University Medical Center (Utrecht, The Netherlands), respectively.

### Mice

All animals used in this study were treated in accordance with Home Office guidance rules under project licences 70/6176 and 70/7078, adhering to the Association for Research in Vision and Ophthalmology (ARVO) Statement for the Use of Animals in Ophthalmic and Vision Research. C57Bl6 mice were used.

### Cell culture

Mouse J774A.1 macrophages were cultured in GlutaMAX^TM^ DMEM (Invitrogen, Carlsbad, CA) with 10% FBS and were maintained at 37°C under 5% CO_2_.

### Porcine RPE cell isolation

Porcine RPE cells were isolated from fresh eyes of young boars obtained from a local slaughterhouse. Briefly, eyes were cut open and the anterior parts were discarded together with the lens and vitreous humour. The posterior eye-cups were filled with PBS and the neuroretina was peeled away and cut off at the optic nerve head. The eye-cups with the exposed RPE layer were incubated with 10× trypsin for 30 min at 37°C, and the cells were resuspended and washed with DMEM containing 10% FBS (Invitrogen, Carlsbad, CA). Cells were maintained at 37°C under 5% CO_2_ in GlutaMAX^TM^ DMEM with 10% FBS. First passage RPE cells were plated onto Transwell® membrane inserts (Corning, New York, NY) and kept in DMEM with 1% FBS for 5–7 days. The transepithelial resistance was measured throughout this time.

### Isolation of POS

POS were isolated from fresh porcine eyes according to an established protocol adapted from Molday and Molday (Molday and Molday, 1987) and further developed and described in detail by Mao and Finnemann (Mao and Finnemann, 2013). Briefly, eyes were cut open and the neuroretina was peeled off and placed in homogenising solution [20% (w/v) sucrose, 20 mM Tris acetate pH 7.2, 2 mM magnesium chloride, 10 mM glucose and 5 mM taurine]. The mixture was vigorously shaken for 2 min, filtered and centrifuged against a continuous sucrose gradient for 1 h at 25,000 rpm in a Beckman SW-27 using a SW32-Ti swing rotor (Brea, CA) at 4°C. The orange band containing the POS was collected, washed three times and finally resuspended in DMEM with 10% FBS.

### Fluid-phase BSA–gold and HRP uptake assay

Monolayers of first-passage primary porcine RPE cells seeded onto polycarbonate Transwell® membrane inserts were incubated from opposite chambers with 10-nm BSA–gold and HRP (7 mg/ml), both diluted in DMEM containing 0.5% BSA, for 2 h at 37°C. Cells were washed thoroughly and processed for conventional electron microscopy as described below.

### Phagocytosis assay

Monolayers of first-passage primary porcine RPE cells seeded onto polycarbonate Transwell® membrane inserts were challenged for 1 h at 37°C with POS in DMEM with 10% FBS (5×10^7^ POS/ml), after POS were subjected to a 10-min sonication step in an ultrasonic bath. After the pulse, cells were washed three times with warm DMEM to remove the unbound POS and chased for 2 h and 4 h. Cells were then washed three times with warm PBS and fixed with 4% paraformaldehyde (PFA) and 0.1% glutaraldehyde in 0.1 M phosphate buffer (pH 7.4) for 3 h at room temperature and processed as described below. For the leupeptin assay, cells were pre-incubated with 5 µg/ml leupeptin (Sigma-Aldrich, St Louis, MO) in DMEM with 10% FBS for 2 h, and the inhibitor was maintained throughout the whole course of the experiment. For the phagocytosis assay with BSA–gold, porcine RPE cells were incubated in the lower chamber with 5-nm BSA–gold diluted in DMEM containing 0.5% BSA for 3 h at 37°C before adding POS to the upper chamber. BSA–gold was maintained throughout the experiment. Cells were chased for 2 h and processed for cryo-immuno-electron microscopy, as described below. The same method was used for the phagocytic assay with mouse macrophages J774A.1.

### Transmission Electron Microscopy

#### Conventional electron microscopy

Colloidal 5-nm and 10-nm gold were coupled to BSA as described previously (Slot and Geuze, 1985). Porcine RPE cells plated onto polycarbonate Transwell® membrane inserts were fixed in 2% PFA and 2% glutaraldehyde in 0.05 M cacodylate buffer for 30 min and post-fixed with 1.5% potassium ferricyanide, 1% osmium tetroxide for 1 h in the dark at 4°C. Cells were then stained with 1% tannic acid in 0.05 µM cacodylate for 40 min, followed by dehydration (70%, 90% and absolute ethanol) and embedding in Epon. Mouse eyes were processed as described previously (Wavre-Shapton et al., 2013). Ultrathin sections were stained with lead citrate and observed on a JEOL 1010 transmission electron microscope and imaged using Gatan Orius SC1000B charge-coupled device camera. POS were spun at 2500 ***g*** for 10 min, resuspended in 2% PFA and 2% glutaraldehyde, fixed for 1 h and processed as described above.

#### Cryo-immuno-electron microscopy

Mouse eyes were fixed in 4% PFA and 0.1% glutaraldehyde in 0.1 M phosphate buffer. The cornea was cut off, the lens was removed and the eye-cup was cut into small pieces. Porcine RPE cells and J7741.A macrophages grown on Transwell® membrane inserts were fixed as above and the inserts were cut into small pieces. Pieces of retina and inserts with cells were embedded in 12% gelatin and infused with 2.3 M sucrose. 70-nm sections were cut at −120°C and collected in a 1∶1 mixture of 2% methylcellulose∶2.3 M sucrose. Labelling was performed as described previously (Slot et al., 1991). Samples were imaged as above and analysed with Gatan Digital Micrograph, Adobe Photoshop and ImageJ softwares.

#### Quantification

To quantify the position of phagosomes by cryo-immuno-electron microscopy, a minimum of 800 µm of RPE length was analysed and the position of the phagosomes was recorded as apical if they were above the tight junctions. All phagosomes below the tight junctions were scored as basal. For quantification of phagosome maturation by cryo-immuno-electron microscopy, the labelling of a minimum of 25 phagosomes was analysed in four eyes. In rhodopsin labelling experiments, phagosomes labelled for 1D4 and RET-P1 were scored as early phagosomes, whereas phagosomes positive for RET-P1 only were scored as late. In the cathepsin D labelling experiment, phagosomes (labelled with RET-P1) that were positive for cathepsin D were scored as phagolysosomes. To determine the density of the labelling in phagosomes, the size of at least 25 phagosomes in four eyes at each time-point was measured and the number of PAG particles coupled to 1D4 was calculated using ImageJ. The density of PAG in the outer segments was also measured in each sample and used to normalise the values obtained in the phagosomes. For quantification of phagosomes in RPE cells, >90 phagosomes for each time-point were scored over three independent experiments.

#### Statistics

To determine the significance of the data, the non-parametric Mann–Whitney test was used throughout. *P*<0.05 was considered statistically significant.

## Supplementary Material

Supplementary Material

## References

[b1] Apodaca G. Katz L. A. Mostov K. E. (1994). Receptor-mediated transcytosis of IgA in MDCK cells is via apical recycling endosomes. J. Cell Biol. 125, 67–86 10.1083/jcb.125.1.67 8138576 PMC2120019

[b2] Blum J. S. Wearsch P. A. Cresswell P. (2013). Pathways of antigen processing. Annu. Rev. Immunol. 31, 443–473 10.1146/annurev-immunol-032712-095910 23298205 PMC4026165

[b3] Bosch E. Horwitz J. Bok D. (1993). Phagocytosis of outer segments by retinal pigment epithelium: phagosome-lysosome interaction. J. Histochem. Cytochem. 41, 253–263 10.1177/41.2.8419462 8419462

[b4] Bright N. A. Gratian M. J. Luzio J. P. (2005). Endocytic delivery to lysosomes mediated by concurrent fusion and kissing events in living cells. Curr. Biol. 15, 360–365 10.1016/j.cub.2005.01.049 15723798

[b5] Burke J. M. Hjelmeland L. M. (2005). Mosaicism of the retinal pigment epithelium: seeing the small picture. Mol. Interv. 5, 241–249 10.1124/mi.5.4.7 16123538

[b6] Chaitin M. H. Hall M. O. (1983). Defective ingestion of rod outer segments by cultured dystrophic rat pigment epithelial cells. Invest. Ophthalmol. Vis. Sci. 24, 812–820 6345445

[b7] D'Cruz P. M. Yasumura D. Weir J. Matthes M. T. Abderrahim H. LaVail M. M. Vollrath D. (2000). Mutation of the receptor tyrosine kinase gene Mertk in the retinal dystrophic RCS rat. Hum. Mol. Genet. 9, 645–651 10.1093/hmg/9.4.645 10699188

[b8] Deguchi J. Yamamoto A. Yoshimori T. Sugasawa K. Moriyama Y. Futai M. Suzuki T. Kato K. Uyama M. Tashiro Y. (1994). Acidification of phagosomes and degradation of rod outer segments in rat retinal pigment epithelium. Invest. Ophthalmol. Vis. Sci. 35, 568–579 8113008

[b9] Desjardins M. Huber L. A. Parton R. G. Griffiths G. (1994). Biogenesis of phagolysosomes proceeds through a sequential series of interactions with the endocytic apparatus. J. Cell Biol. 124, 677–688 10.1083/jcb.124.5.677 8120091 PMC2119957

[b10] Duclos S. Diez R. Garin J. Papadopoulou B. Descoteaux A. Stenmark H. Desjardins M. (2000). Rab5 regulates the kiss and run fusion between phagosomes and endosomes and the acquisition of phagosome leishmanicidal properties in RAW 264.7 macrophages. J. Cell Sci. 113, 3531–3541 10984443 10.1242/jcs.113.19.3531

[b11] Esteve-Rudd J. Lopes V. S. Jiang M. Williams D. S. (2014). In vivo and in vitro monitoring of phagosome maturation in retinal pigment epithelium cells. Adv. Exp. Med. Biol. 801, 85–90 10.1007/978-1-4614-3209-8_11 24664684

[b12] Feng W. Yasumura D. Matthes M. T. LaVail M. M. Vollrath D. (2002). Mertk triggers uptake of photoreceptor outer segments during phagocytosis by cultured retinal pigment epithelial cells. J. Biol. Chem. 277, 17016–17022 10.1074/jbc.M107876200 11861639

[b13] Finnemann S. C. (2003). Focal adhesion kinase signaling promotes phagocytosis of integrin-bound photoreceptors. EMBO J. 22, 4143–4154 10.1093/emboj/cdg416 12912913 PMC175805

[b14] Flannagan R. S. Jaumouillé V. Grinstein S. (2012). The cell biology of phagocytosis. Annu. Rev. Pathol. 7, 61–98 10.1146/annurev-pathol-011811-132445 21910624

[b15] Fratti R. A. Backer J. M. Gruenberg J. Corvera S. Deretic V. (2001). Role of phosphatidylinositol 3-kinase and Rab5 effectors in phagosomal biogenesis and mycobacterial phagosome maturation arrest. J. Cell Biol. 154, 631–644 10.1083/jcb.200106049 11489920 PMC2196432

[b16] Futter C. E. Gibson A. Allchin E. H. Maxwell S. Ruddock L. J. Odorizzi G. Domingo D. Trowbridge I. S. Hopkins C. R. (1998). In polarized MDCK cells basolateral vesicles arise from clathrin-gamma-adaptin-coated domains on endosomal tubules. J. Cell Biol. 141, 611–623 10.1083/jcb.141.3.611 9566963 PMC2132747

[b17] Futter C. E. Ramalho J. S. Jaissle G. B. Seeliger M. W. Seabra M. C. (2004). The role of Rab27a in the regulation of melanosome distribution within retinal pigment epithelial cells. Mol. Biol. Cell 15, 2264–2275 10.1091/mbc.E03-10-0772 14978221 PMC404021

[b18] Ghosh P. Dahms N. M. Kornfeld S. (2003). Mannose 6-phosphate receptors: new twists in the tale. Nat. Rev. Mol. Cell Biol. 4, 202–213 10.1038/nrm1050 12612639

[b19] Gibbs D. Kitamoto J. Williams D. S. (2003). Abnormal phagocytosis by retinal pigmented epithelium that lacks myosin VIIa, the Usher syndrome 1B protein. Proc. Natl. Acad. Sci. USA 100, 6481–6486 10.1073/pnas.1130432100 12743369 PMC164472

[b20] Gibbs D. Azarian S. M. Lillo C. Kitamoto J. Klomp A. E. Steel K. P. Libby R. T. Williams D. S. (2004). Role of myosin VIIa and Rab27a in the motility and localization of RPE melanosomes. J. Cell Sci. 117, 6473–6483 10.1242/jcs.01580 15572405 PMC2942070

[b21] Gibbs D. Diemer T. Khanobdee K. Hu J. Bok D. Williams D. S. (2010). Function of MYO7A in the human RPE and the validity of shaker1 mice as a model for Usher syndrome 1B. Invest. Ophthalmol. Vis. Sci. 51, 1130–1135 10.1167/iovs.09-4032 19643958 PMC2868451

[b22] Gordiyenko N. V. Fariss R. N. Zhi C. MacDonald I. M. (2010). Silencing of the CHM gene alters phagocytic and secretory pathways in the retinal pigment epithelium. Invest. Ophthalmol. Vis. Sci. 51, 1143–1150 10.1167/iovs.09-4117 19741243 PMC2868448

[b23] Gutierrez M. G. (2013). Functional role(s) of phagosomal Rab GTPases. Small GTPases 4, 148–158 10.4161/sgtp.25604 24088602 PMC3976971

[b24] Harrison R. E. Bucci C. Vieira O. V. Schroer T. A. Grinstein S. (2003). Phagosomes fuse with late endosomes and/or lysosomes by extension of membrane protrusions along microtubules: role of Rab7 and RILP. Mol. Cell. Biol. 23, 6494–6506 10.1128/MCB.23.18.6494-6506.2003 12944476 PMC193691

[b25] Herman K. G. Steinberg R. H. (1982a). Phagosome degradation in the tapetal retinal pigment epithelium of the opossum. Invest. Ophthalmol. Vis. Sci. 23, 291–304 7107157

[b26] Herman K. G. Steinberg R. H. (1982b). Phagosome movement and the diurnal pattern of phagocytosis in the tapetal retinal pigment epithelium of the opossum. Invest. Ophthalmol. Vis. Sci. 23, 277–290 7107156

[b27] Hirst J. Futter C. E. Hopkins C. R. (1998). The kinetics of mannose 6-phosphate receptor trafficking in the endocytic pathway in HEp-2 cells: the receptor enters and rapidly leaves multivesicular endosomes without accumulating in a prelysosomal compartment. Mol. Biol. Cell 9, 809–816 10.1091/mbc.9.4.809 9529379 PMC25308

[b28] Horwitz M. A. Maxfield F. R. (1984). Legionella pneumophila inhibits acidification of its phagosome in human monocytes. J. Cell Biol. 99, 1936–1943 10.1083/jcb.99.6.1936 6501409 PMC2113576

[b29] Katz M. L. Robison W. G. Jr (1984). Age-related changes in the retinal pigment epithelium of pigmented rats. Exp. Eye Res. 38, 137–151 10.1016/0014-4835(84)90098-8 6714331

[b30] Kennedy C. J. Rakoczy P. E. Constable I. J. (1995). Lipofuscin of the retinal pigment epithelium: a review. Eye (Lond.) 9, 763–771 10.1038/eye.1995.192 8849547

[b31] Kielian M. C. Cohn Z. A. (1980). Phagosome-lysosome fusion. Characterization of intracellular membrane fusion in mouse macrophages. J. Cell Biol. 85, 754–765 10.1083/jcb.85.3.754 7391139 PMC2111457

[b32] Law A. L. Ling Q. Hajjar K. A. Futter C. E. Greenwood J. Adamson P. Wavre-Shapton S. T. Moss S. E. Hayes M. J. (2009). Annexin A2 regulates phagocytosis of photoreceptor outer segments in the mouse retina. Mol. Biol. Cell 20, 3896–3904 10.1091/mbc.E08-12-1204 19587120 PMC2735488

[b33] Lopes V. S. Wasmeier C. Seabra M. C. Futter C. E. (2007). Melanosome maturation defect in Rab38-deficient retinal pigment epithelium results in instability of immature melanosomes during transient melanogenesis. Mol. Biol. Cell 18, 3914–3927 10.1091/mbc.E07-03-0268 17671165 PMC1995718

[b34] Mao Y. Finnemann S. C. (2013). Analysis of photoreceptor outer segment phagocytosis by RPE cells in culture. Methods Mol. Biol. 935, 285–295 10.1007/978-1-62703-080-9_20 23150376 PMC3590840

[b35] Molday R. S. MacKenzie D. (1985). Inhibition of monoclonal antibody binding and proteolysis by light-induced phosphorylation of rhodopsin. Biochemistry 24, 776–781 10.1021/bi00324a036 2581604

[b36] Molday L. L. Molday R. S. (1987). Glycoproteins specific for the retinal rod outer segment plasma membrane. Biochim. Biophys. Acta 897, 335–340 10.1016/0005-2736(87)90430-5 2434131

[b37] Nandrot E. F. Kim Y. Brodie S. E. Huang X. Sheppard D. Finnemann S. C. (2004). Loss of synchronized retinal phagocytosis and age-related blindness in mice lacking alphavbeta5 integrin. J. Exp. Med. 200, 1539–1545 10.1084/jem.20041447 15596525 PMC2211990

[b38] Nandrot E. F. Anand M. Almeida D. Atabai K. Sheppard D. Finnemann S. C. (2007). Essential role for MFG-E8 as ligand for alphavbeta5 integrin in diurnal retinal phagocytosis. Proc. Natl. Acad. Sci. USA 104, 12005–12010 10.1073/pnas.0704756104 17620600 PMC1924559

[b39] Petit C. (2001). Usher syndrome: from genetics to pathogenesis. Annu. Rev. Genomics Hum. Genet. 2, 271–297 10.1146/annurev.genom.2.1.271 11701652

[b40] Rakoczy P. E. Sarks S. H. Daw N. Constable I. J. (1999). Distribution of cathepsin D in human eyes with or without age-related maculopathy. Exp. Eye Res. 69, 367–374 10.1006/exer.1999.0700 10504270

[b41] Rakoczy P. E. Zhang D. Robertson T. Barnett N. L. Papadimitriou J. Constable I. J. Lai C. M. (2002). Progressive age-related changes similar to age-related macular degeneration in a transgenic mouse model. Am. J. Pathol. 161, 1515–1524 10.1016/S0002-9440(10)64427-6 12368224 PMC1867306

[b42] Raposo G. Marks M. S. (2002). The dark side of lysosome-related organelles: specialization of the endocytic pathway for melanosome biogenesis. Traffic 3, 237–248 10.1034/j.1600-0854.2002.030401.x 11929605

[b43] Raposo G. Tenza D. Murphy D. M. Berson J. F. Marks M. S. (2001). Distinct protein sorting and localization to premelanosomes, melanosomes, and lysosomes in pigmented melanocytic cells. J. Cell Biol. 152, 809–824 10.1083/jcb.152.4.809 11266471 PMC2195785

[b44] Renfrew C. A. Hubbard A. L. (1991). Sequential processing of epidermal growth factor in early and late endosomes of rat liver. J. Biol. Chem. 266, 4348–4356 1671862

[b45] Slot J. W. Geuze H. J. (1985). A new method of preparing gold probes for multiple-labeling cytochemistry. Eur. J. Cell Biol. 38, 87–93 4029177

[b46] Slot J. W. Geuze H. J. Gigengack S. Lienhard G. E. James D. E. (1991). Immuno-localization of the insulin regulatable glucose transporter in brown adipose tissue of the rat. J. Cell Biol. 113, 123–135 10.1083/jcb.113.1.123 2007617 PMC2288909

[b47] Strauss O. (2005). The retinal pigment epithelium in visual function. Physiol. Rev. 85, 845–881 10.1152/physrev.00021.2004 15987797

[b48] Thompson D. A. Gal A. (2003). Vitamin A metabolism in the retinal pigment epithelium: genes, mutations, and diseases. Prog. Retin. Eye Res. 22, 683–703 10.1016/S1350-9462(03)00051-X 12892646

[b49] Thumann G. Bartz-Schmidt K. U. Kociok N. Heimann K. Schraemeyer U. (1999). Ultimate fate of rod outer segments in the retinal pigment epithelium. Pigment Cell Res. 12, 311–315 10.1111/j.1600-0749.1999.tb00764.x 10541040

[b50] Vergne I. Chua J. Singh S. B. Deretic V. (2004). Cell biology of mycobacterium tuberculosis phagosome. Annu. Rev. Cell Dev. Biol. 20, 367–394 10.1146/annurev.cellbio.20.010403.114015 15473845

[b51] Vieira O. V. Bucci C. Harrison R. E. Trimble W. S. Lanzetti L. Gruenberg J. Schreiber A. D. Stahl P. D. Grinstein S. (2003). Modulation of Rab5 and Rab7 recruitment to phagosomes by phosphatidylinositol 3-kinase. Mol. Cell. Biol. 23, 2501–2514 10.1128/MCB.23.7.2501-2514.2003 12640132 PMC150733

[b52] Wavre-Shapton S. T. Tolmachova T. Lopes da Silva M. Futter C. E. Seabra M. C. (2013). Conditional ablation of the choroideremia gene causes age-related changes in mouse retinal pigment epithelium. PLoS ONE 8, e57769 10.1371/journal.pone.0057769 23460904 PMC3584022

[b53] Yamada T. Hara S. Tamai M. (1990). Immunohistochemical localization of cathepsin D in ocular tissues. Invest. Ophthalmol. Vis. Sci. 31, 1217–1223 2194988

[b54] Young R. W. Bok D. (1969). Participation of the retinal pigment epithelium in the rod outer segment renewal process. J. Cell Biol. 42, 392–403 10.1083/jcb.42.2.392 5792328 PMC2107669

[b55] Zhang D. Lai M. C. Constable I. J. Rakoczy P. E. (2002). A model for a blinding eye disease of the aged. Biogerontology 3, 61–66 10.1023/A:1015259413857 12014844

[b56] Zimmerman W. F. Godchaux W. 3rd Belkin M. (1983). The relative proportions of lysosomal enzyme activities in bovine retinal pigment epithelium. Exp. Eye Res. 36, 151–158 10.1016/0014-4835(83)90098-2 6825729

