# Supplementary material for: Phagosome maturation during endosome interaction revealed by partial rhodopsin processing in retinal pigment epithelium
Source: J Cell Sci. 2014 Sep 1;127(17):3852–61. doi: 10.1242/jcs.154757 (PMC4150067; doi:10.1242/jcs.154757)
Supplement: Supplementary Material [file supp_127_17_3852__index.html]

Phagosome maturation during endosome interaction revealed by partial rhodopsin processing in retinal pigment epithelium — Supplementary Material 

# Phagosome maturation during endosome interaction revealed by partial rhodopsin processing in retinal pigment epithelium

## JCS154757 Supplementary Material

**Files in this Data Supplement:**

- **Supplementary Material**
