# Supplementary material for: Phagosome maturation during endosome interaction revealed by partial rhodopsin processing in retinal pigment epithelium
Source: J Cell Sci. 2014 Sep 1;127(17):3852–61. doi: 10.1242/jcs.154757 (PMC4150067; doi:10.1242/jcs.154757)
Supplement: Supplementary Material [file supp_127.17.3852_JCS154757.pdf]

SUPPLEMENTARY MATERIAL

Supplementary Figures

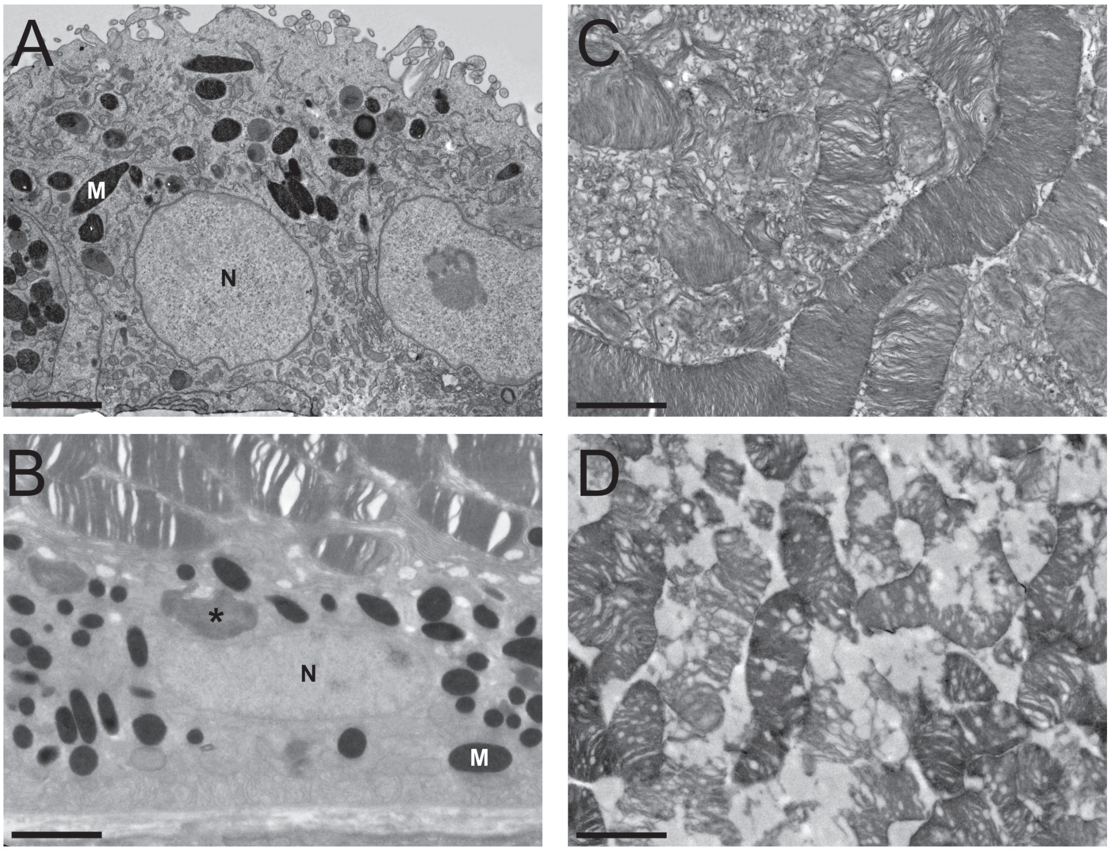

Figure S1

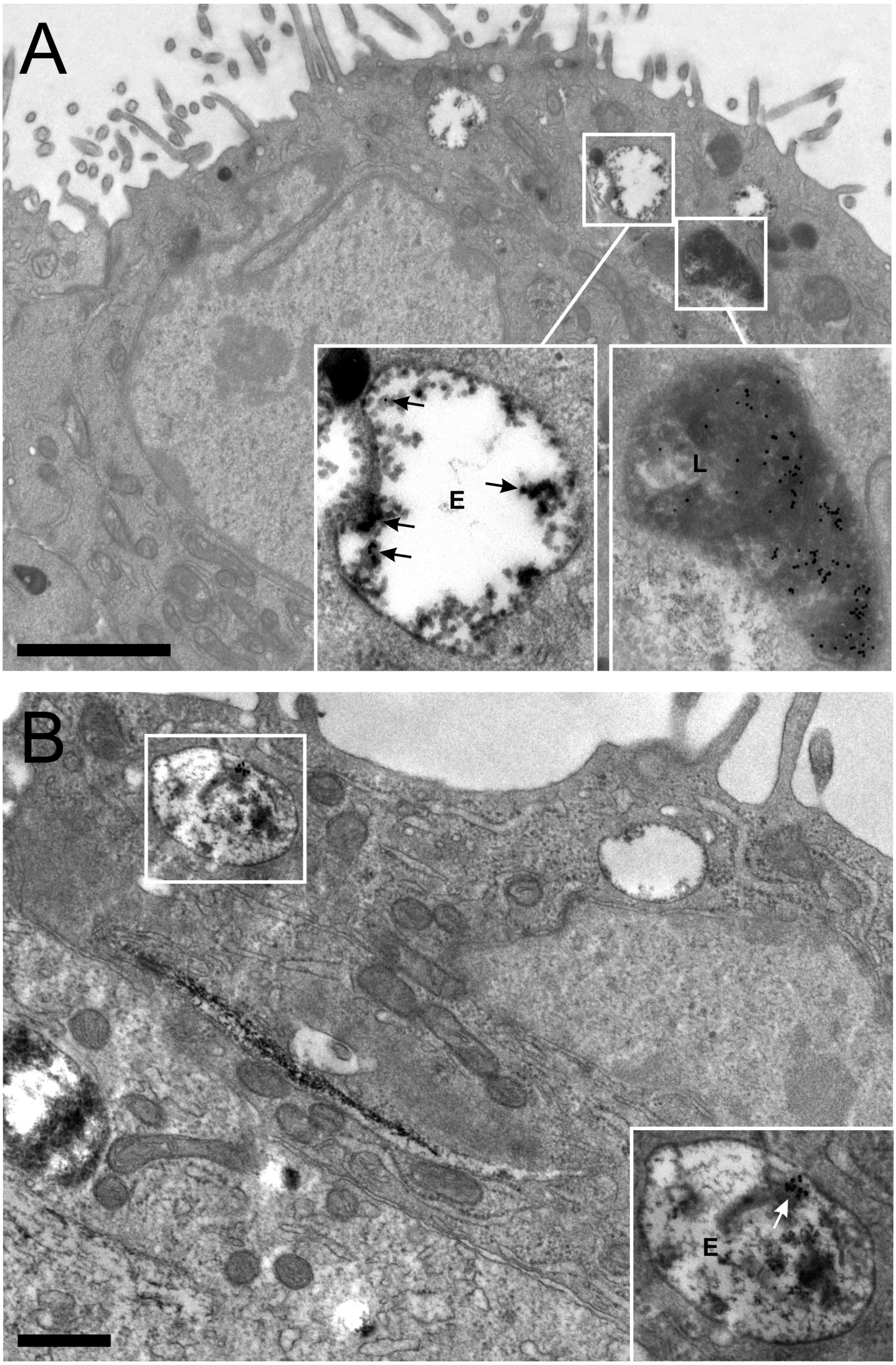

Figure S2

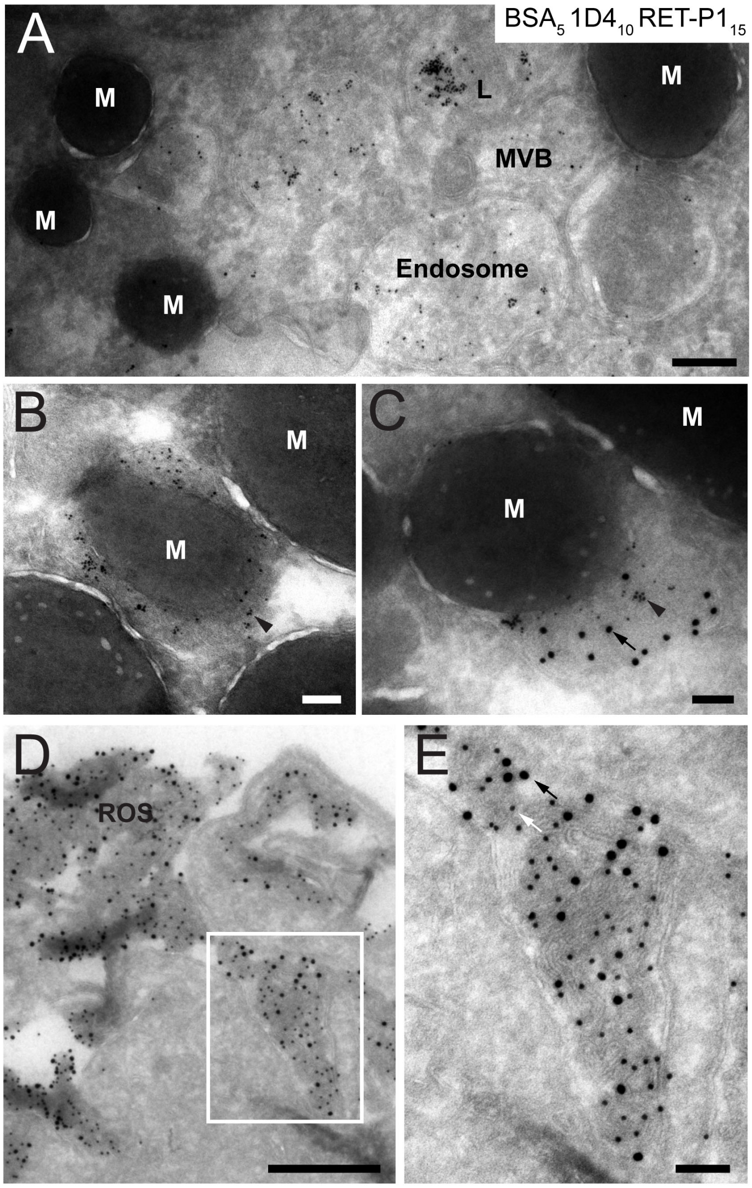

Figure S3

Supplementary Figure Legends

Fig. S1. Characterisation of porcine RPE and POS. A) Primary porcine RPE cells were seeded onto Transwell® membrane inserts, cultured for 7-10 days and analysed by conventional EM. Note the presence of apical processes, melanosomes close to the apical region and mitochondria around the basolateral border. B) Wild type mouse retinal section for comparison. C) Non-sonicated POS and D) POS sonicated for 10 min. Note that after sonication the isolated POS resemble the size of apical phagosomes in the retinal section in B (asterisk). Scale bar, 2µm. N, nucleus; M, melanosome.

Fig S2: Fluid phase probes added to opposite chambers meet in a common endocytic compartment in primary porcine RPE cells. Monolayers of primary porcine RPE cells on Transwell® membrane inserts were incubated from opposite chambers with BSA-gold and fluid phase HRP (7 mg/mL) for 2 h at 37°C and were analysed by conventional EM. A) Cells incubated with BSA-gold in the apical chamber and HRP in the basal chamber. Insets show endosomal and lysosomal compartments. B) Cells incubated with HRP in the apical chamber and BSA-gold in the basal chamber. Inset shows an endosomal compartment. Note that both probes meet in a common endocytic compartment within the cell that is electron luscent and contains monodisperse gold particles (black arrows in A and white in B), as well as co-localising in electron dense lysosomal compartments. Scale bars, 2 µm in A and 0.5 µm in B. E, endosome; L, lysosome.

Fig. S3: The endocytic pathway in porcine RPE cells can be filled with 5nm BSA-gold. Monolayers of primary porcine RPE cells on Transwell® membrane insert were incubated in the basal chamber with 5 nm BSA-gold for 3 h prior addition of POS and BSA-gold was maintained throughout the experiment. Cells were then challenged with POS from the apical chamber for 1 h, washed to remove unbound POS, chased for 2 h at 37°C and processed for cryo-immunoEM. Ultrathin sections were double-labelled for rhodopsin with antibodies against C-terminal epitope (1D4, PAG 10 nm) and N-terminal epitope (RET-P1, PAG 15 nm). A) Endosomes, lysosomes (L) and multivesicular body (MVB) containing 5 nm BSA-gold. Note that the gold particles aggregate in the lysosomes. Most melanosomes (M) do not contain any 5 nm BSA-gold. B) Melanosome associated with a 5 nm BSA-gold compartment, C). Melanosome associated with a 5 nm BSA-gold (black arrowhead) and PAG 15 nm

(black arrow, RET-P1) positive compartment. D and E) POS being engulfed by porcine RPE cells do not contain any 5 nm BSA-gold (white box in D is enlarged in E).

Scale bars, 200 nm in A, 100 nm in B, C and E, 500 nm in D. M, melanosomes; MVB, multivesicular body; POS, photoreceptor outer segment. Black and white arrows indicate rhodopsin labelling with RET-P1 and 1D4 respectively; black arrowheads indicate 5 nm BSA-gold.
